# Supplementary material for: In the face of climate change and exhaustive exercise: the physiological response of an important recreational fish species
Source: R Soc Open Sci. 2020 Mar 25;7(3):200049. doi: 10.1098/rsos.200049 (PMC7137940; doi:10.1098/rsos.200049)
Supplement: Supplementary Tables and Figures [file rsos200049supp1.docx]

**In the face of climate change and exhaustive exercise: The physiological response of an important recreational fish species**

Daniel P. Crear, Fisheries Science Department, Virginia Institute of Marine Science, William & Mary, Gloucester Point, VA 23062, USA

Rich W. Brill, Fisheries Science Department, Virginia Institute of Marine Science, William & Mary, Gloucester Point, VA 23062, USA

Lauren M. L. Averilla, Biology Department, William & Mary, Williamsburg, VA, 23185

Sara C. Meakem, Department of Kinesiology & Health Sciences, William & Mary, Williamsburg, VA, 23185

Kevin C. Weng, Fisheries Science Department, Virginia Institute of Marine Science, William & Mary, Gloucester Point, VA 23062, USA

**Royal Society Open Science**

**Supplementary Data**

Supplementary Table 1. ∆BIC table of candidate models to describe metabolic rate of cobia under normoxic conditions. All models included heterogeneity and a correlation structure of AR1. The random effect of Animal ID is indicated by (1|Animal_ID). To meet model assumptions, metabolic rate was log transformed.

| Model # | Models describing log (metabolic rate) | DF | ∆BIC |
| --- | --- | --- | --- |
| 1 | (1\|Animal_ID) + VRMG + Test_Temp + TL + Trial_Time | 11 | 0 |
| 2 | (1\|Animal_ID) + (VRMG * TL) + Test_Temp + Time_Trial | 12 | 7.1 |
| 3 | (1\|Animal_ID) + VRMG + Test_Temp + Time_Trial | 10 | 8.3 |
| 4 | (1\|Animal_ID) + (VRMG * Test_Temp) + TL + Time_Trial | 13 | 13.6 |
| 5 | (1\|Animal_ID) + VRMG + Test_Temp + TL | 10 | 145.5 |
| 6 | (1\|Animal_ID) + VRMG + Test_Temp | 9 | 152.1 |

Supplementary Table 2. ∆BIC table of candidate models to describe metabolic rate of cobia under hypoxic conditions. All models included heterogeneity and a correlation structure of AR1. The random effect of Animal ID is indicated by (1|Animal_ID).

| Model # | Models describing metabolic rate | DF | \| ∆BIC \| \| --- \| |
| --- | --- | --- | --- | --- |
| 1 | (1\|Animal_ID) + (VRMG * O2) + Test_Temp + TL | 12 | 0 |
| 2 | (1\|Animal_ID) + VRMG + O2 + Test_Temp + TL | 11 | 2.3 |
| 3 | (1\|Animal_ID) + (VRMG * TL) + Test_Temp + O2 | 12 | 8.3 |
| 4 | (1\|Animal_ID) + VRMG + O2 + Test_Temp | 10 | 8.9 |
| 5 | (1\|Animal_ID) + (VRMG * Test_Temp) + TL + O2 | 13 | 12.2 |
| 6 | (1\|Animal_ID) + VRMG + (Test_Temp * O2) + TL | 13 | 13 |


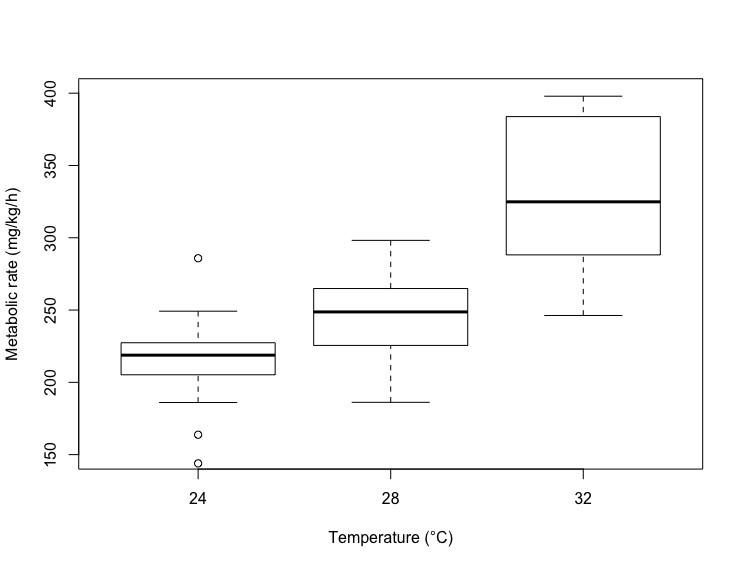


Supplementary Figure 1. Boxplots of raw values of maximum metabolic rate of cobia tested at 24, 28, and 32°C.


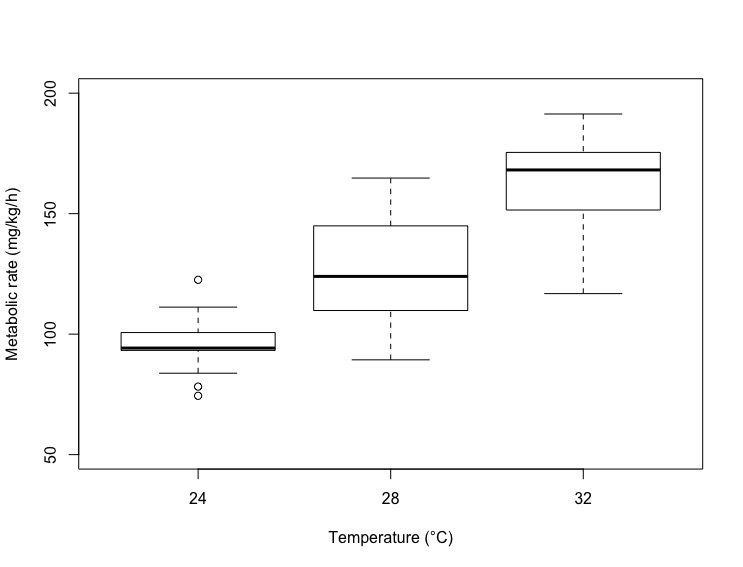


Supplementary Figure 2. Boxplots of raw values of standard metabolic rate of cobia tested at 24, 28, and 32°C.


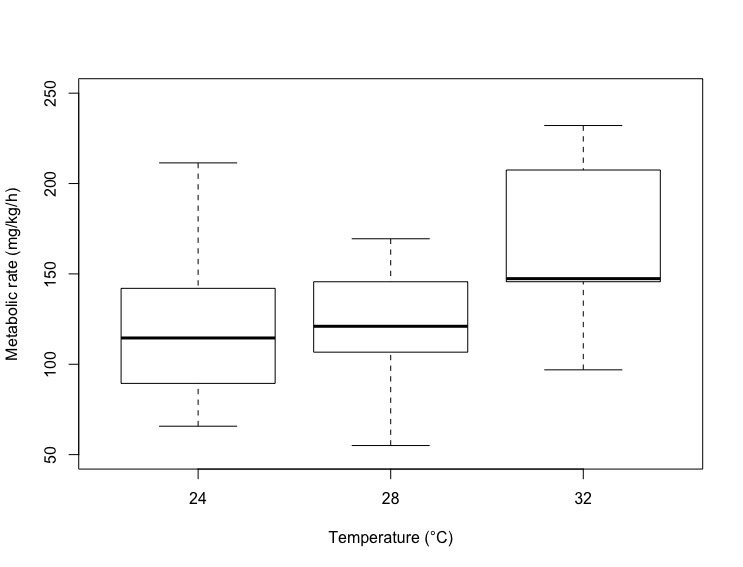


Supplementary Figure 3. Boxplots of raw values of aerobic scope of cobia tested at 24, 28, and 32°C.


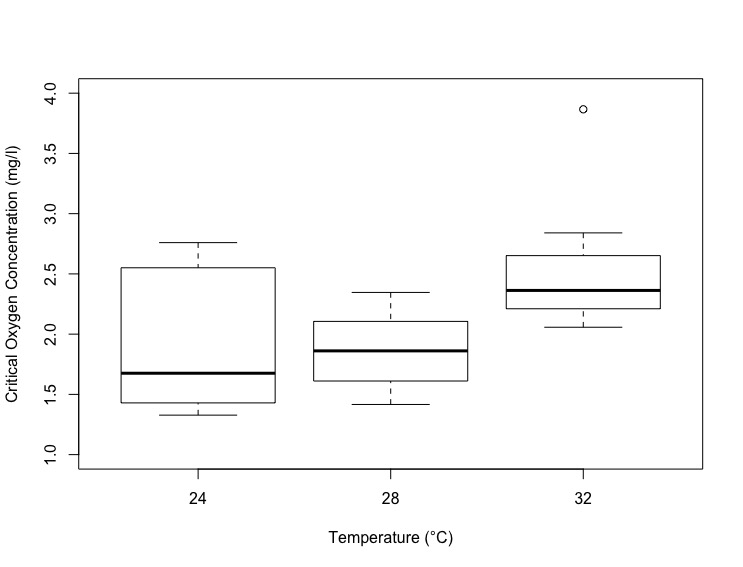


Supplementary Figure 4. Boxplots of raw values of C_crit_ of cobia tested at 24, 28, and 32°C.
